# Circadian peak dopaminergic activity response at the biological clock pacemaker (suprachiasmatic nucleus) area mediates the metabolic responsiveness to a high‐fat diet

**DOI:** 10.1111/jne.12563

**Published:** 2018-01-23

**Authors:** S. Luo, Y. Zhang, M. Ezrokhi, Y. Li, T.‐H. Tsai, A. H. Cincotta

**Affiliations:** ^1^ VeroScience LLC Tiverton RI USA

**Keywords:** circadian, diabetes, dopamine, insulin sensitivity, suprachiasmatic nuclei

## Abstract

Among vertebrate species of the major vertebrate classes in the wild, a seasonal rhythm of whole body fuel metabolism, oscillating from a lean to obese condition, is a common biological phenomenon. This annual cycle is driven in part by annual changes in the circadian dopaminergic signalling at the suprachiasmatic nuclei (SCN), with diminution of circadian peak dopaminergic activity at the SCN facilitating development of the seasonal obese insulin‐resistant condition. The present study investigated whether such an ancient circadian dopamine‐SCN activity system for expression of the seasonal obese, insulin‐resistant phenotype may be operative in animals made obese amd insulin resistant by high‐fat feeding and, if so, whether reinstatement of the circadian dopaminergic peak at the SCN would be sufficient to reverse the adverse metabolic impact of the high‐fat diet without any alteration of caloric intake. First, we identified the supramammillary nucleus as a novel site providing the majority of dopaminergic neuronal input to the SCN. We further identified dopamine D2 receptors within the peri‐SCN region as being functional in mediating SCN responsiveness to local dopamine. In lean, insulin‐sensitive rats, the peak in the circadian rhythm of dopamine release at the peri‐SCN coincided with the daily peak in SCN electrophysiological responsiveness to local dopamine administration. However, in rats made obese and insulin resistant by high‐fat diet (HFD) feeding, these coincident circadian peak activities were both markedly attenuated or abolished. Reinstatement of the circadian peak in dopamine level at the peri‐SCN by its appropriate circadian‐timed daily microinjection to this area (but not outside this circadian time‐interval) abrogated the obese, insulin‐resistant condition without altering the consumption of the HFD. These findings suggest that the circadian peak of dopaminergic activity at the peri‐SCN/SCN is a key modulator of metabolism and the responsiveness to adverse metabolic consequences of HFD consumption.

## 1 INTRODUCTION

In the wild, vertebrate species, from teleosts to mammals, exhibit marked annual cycles of metabolism, oscillating between obese and lean conditions during particular seasons of the year. The seasonal obese condition is coupled with the hyperinsulinaemic, insulin‐resistant state similar to the human metabolic syndrome.^1^, ^2^ This obese insulin‐resistant state imparts a survival advantage to the animal during an ensuing/existing season of low/no food (particularly glucose) availability. The available evidence suggests that, under such circumstances, insulin resistance facilitates increased endogenous glucose production to fuel central nervous system metabolism at the same time as the peripheral tissues increase their utilisation of stored fat.^1^, ^2^ Importantly, among the seasonal species studied, the shift from the obese, insulin‐resistant state to the lean, insulin‐sensitive condition does not require a change in photoperiod and can occur without any change in food consumption.^1^, ^2^, ^3^, ^4^ Investigations of such seasonal metabolism under controlled laboratory conditions have identified important roles for the endogenous circadian biological clock system, particularly the circadian rhythm of dopamine (DA) input signalling to the biological clock area in the hypothalamus (the suprachiasmatic nuclei; SCN) in the regulation of seasonal metabolism and of seasonality itself (defined as a change in physiological responsiveness to a particular photoperiod at a particular time of year).^5^, ^6^ Daily systemic injections of l‐3,4‐dihydroxyphenylalanine (l‐DOPA, a precursor for dopamine synthesis) to animals that are maintained under constant light conditions can induce the entire seasonal repertoire of physiological/metabolic states in representative species of fish, birds and mammals within just a few days as a function of the circadian time of day of its administration relative to the time of day of daily injections of 5‐hydroxytryptophan (a precursor for serotonin synthesis) irrespective of the actual time of year of such treatment.^7^, ^8^, ^9^, ^10^ The temporal phase relationships between the circadian peaks in dopaminergic and serotonergic neural activities at the SCN area differ with seasonal condition, even in animals held under the same photoperiod at the same time of year (seasonality) (eg, summer/autumn animals in the glucose tolerant condition on long daily photoperiods [>14 hours of light; termed photosensitive] vs summer/autumn animals in the glucose intolerant condition on the same long daily photoperiods [termed photorefractory]).^5^ At the same time as seasonal animals transition from the insulin‐sensitive, glucose tolerant state to the insulin‐resistant, glucose intolerant state, when maintained on the same daily photoperiod, there is a marked two‐thirds reduction in the circadian peak dopaminergic input activity at the area of the SCN.^5^ Moreover, specific lesion of these SCN‐area dopaminergic neurones in seasonally lean, insulin‐sensitive animals results in the obese insulin‐resistant state that cannot be explained by any change in food consumption.^6^ It is suggested that the phase relationship between these two (dopaminergic and serotonergic) circadian neural oscillation input signals to the SCN regulates (circadian) output activities from the clock that modulate physiological status based upon their effect to synchronise the phase relationships of multiple peripheral circadian stimulus (eg, insulin) and response (eg, hepatic lipogenic responsiveness to insulin) rhythms.^1^, ^2^ Relevant to these findings are the independent observations of tyrosine‐hydroxylase immunopositive (TH+) fibres observed in the SCN area (within the structure and around its perimeter) of perinatal rodents including Syrian hamsters, Siberian hamsters and rats that diminish in density (although they are still present) within the SCN but are still relatively prominent in the peri‐SCN region of the adult.^11^, ^12^, ^13^, ^14^, ^15^, ^16^, ^17^, ^18^ The peri‐SCN TH+ fibres were also dopamine β‐hydroxylase negative and/or aromatic amino acid decarboxylase positive, suggesting a dopaminergic neuronal function.^11^, ^12^ The origin(s) of these TH+ fibres, however, have not been identified and could represent short local (inter)neurones, as well as projections from other anatomical sites, with this being one focus of the present study (see below).

We postulated that this ancient circadian control system for whole body regulation of fuel metabolism may modulate the sensitivity of the body to the obesity/insulin resistance‐inducing effects of a high‐fat diet (HFD) and, as such, play an important role in regulating the metabolic syndrome‐inducing effects of the westernised diet of modern man. This clock system for the regulation of metabolism is sensitive to seasonal changes in nutrient quality (eg, changes in natural flora and fauna cycles)^1^ and HFDs have been demonstrated to reduce striatal (mesolimbic) dopamine levels or dopamine receptor availability.^19^, ^20^, ^21^, ^22^, ^23^, ^24^, ^25^, ^26^ We therefore postulated that, in animals made obese and insulin resistant by HFD feeding, the circadian peak of dopaminergic input activity to the SCN area would be diminished and also that local SCN restoration of this circadian dopaminergic input activity would be sufficient to reverse the metabolic syndrome‐inducing impact of the high fat diet upon metabolism. As such, investigations were undertaken aiming to (i) identify an anatomical source of dopaminergic projections to the SCN and/or to peri‐SCN neurones (neurones within approximately 0.25‐0.4 mm of the SCN lateral border) that may communicate with the SCN and (ii) determine the circadian activity of such dopaminergic circuitry and what, if any, is the influence of the obese‐insulin‐resistant state induced by HFD feeding upon such circadian dopaminergic input‐SCN communication activity. Most importantly, a second series of follow‐on studies investigated the impact of circadian‐timed, dopamine local administration to the identified dopaminergic neurone terminals at the peri‐SCN region upon metabolism of animals made obese/insulin resistant by HFD feeding.

## 2 MATERIALS AND METHODS

### 2.1 Experimental animals

Laboratory rats (Sprague‐Dawley [SD], Taconic Biosciences, Hudson, NY, USA, or spontaneously hypertensive rats [SHR], Charles River Laboratories, Wilmington, MA, USA) (10‐12 weeks of age) were housed individually and maintained in a temperature controlled room on 14 hour daily photoperiods (14 : 10 hour light/dark [LD] cycle) during all experimental study periods. Animals were allowed to feed either a HFD (60% of calories from fat; d12492; Research Diets Inc., New Brunswick, NJ, USA) or regular rodent chow (RC) diet (18% of calories from fat; Envigo Teklad 2018; Envigo Teklad, Indianapolis, IN, USA) and to drink ad libitum throughout the study period. All animal experiments were conducted in accordance with the National Institutes of Health Guide for the Care and Use of Experimental Animals and also with the protocols approved by the Institutional Animal Care and Use Committee of VeroScience, LLC.

### 2.2 Fluorogold retrograde tracing and fluorogold/tyrosine hydroxylase (TH) dual immunohistochemistry

Female SD rats weighing 225‐275 g, maintained under LD 14 : 10 daily photoperiod and fed a RC diet were anaesthetised with ketamine/xylazine (60/5 mg kg^‐1^ body weight, i.p.) and secured on a stereotaxic apparatus. Retrograde tracer fluorogold (FG) (4% in 20 nL of saline) was microinjected using a glass micropipette (inner tip diameter 20‐25 μm) driven by a Nanoject injector (Drummond Scientific Company, Broomall, PA, USA) into the SCN area (1.3 mm posterior to the Bregma, 0.2 mm from midline and 9.3 mm below the skull). Ten days after FG microinjection, the rats were sacrificed under anesthesia by transcardiac perfusion with 4% paraformaldehyde. Brains were post‐fixed in 4% paraformaldehyde overnight and cryoprotected in 30% sucrose/phosphate‐buffered saline (pH 7.4). Free floating coronal brain sections (30 μm) were cut from frozen brains on a cryostat and sequentially collected in a freezing solution containing 30% ethylene glycol, 25% glycerol and 0.05 m phosphate buffer saline (pH 7.4) and kept at −20°C until use. For the dual‐labelling of fluorogold and TH, brain sections were first treated with 3% hydrogen peroxide for 10 minutes to quench the endogenous peroxidase activity followed by 1 hour of incubation in animal‐free blocker (Vector Laboratories, Burlingame, CA, USA). Then, the sections were immunolabelled at 4°C overnight with a rabbit polyclonal antiserum for FG (dilution 1:3000; AB153; Millipore, Billerica, MA, USA) followed by 1 hour of incubation at room temperature with a goat anti‐rabbit biotinylated secondary antiserum (dilution 1:500; Vector Laboratories). The FG immunoreactivity was amplified by an avidin‐biotin complex (ABC) system and revealed as dark brown 3,3′‐diaminobenzidine (DAB) punctates. Following thorough washing, the sections were re‐blocked with animal‐free blocker and then sequentially incubated with the mouse monoclonal antiserum for TH (dilution 1:1000; MAB5280; Millipore) at 4°C overnight and a horse anti‐mouse biotinylated secondary antiserum (dilution 1:500; Vector Laboratories) for 1 hour at room temperature. Following amplification with the ABC system, the TH immunoreactivities were revealed as diffuse blue‐grey staining in the cytoplasm (Vector SG peroxidase substrate kit, SK‐4700; Vector Laboratories). The dual‐labelled cells were examined under a light microscope (Nikon, Tokyo, Japan). Images were captured by a digital camera and processed using photoshop (Adobe Systems, San Jose, CA, USA).

### 2.3 In vivo microdialysis

Animals (SD rats) utilised in microdialysis studies were anaesthetised with ketamine/xylazine (80/12 mg kg^‐1^ body weight, i.p.) and mounted on a stereotaxic apparatus (David Kopf Instruments, Tujunga, CA, USA) for implantation of microdialysis probes. In separate studies, a 30‐gauge stainless steel guide cannula (CMA Microdialysis, Holliston, MA, USA) was permanently implanted aimed either at the top of the right SCN at coordinates 1.3 mm posterior to bregma, 0.25 right lateral to the midsagittal suture and 8.3 mm ventral to the dura, with the incisor bar set 3 mm below the interaural line or at the top of the ventromedial hypothalamus (VMH) at coordinates 2.6 mm posterior to bregma, 0.6 mm right lateral to the midsagittal suture and 9 mm ventral to the dura. The guide cannula was anchored firmly to the skull with three stainless‐steel screws and cemented in place with dental acrylic. Animals were allowed 1 week to recover prior to the initiation of microdialysis experimentation. During microdialysis, each animal was placed in an acrylic bowl with free access to food and water. A 32‐gauge dialysis probe with a 1‐mm long tip of semi‐permeable membrane (20 000 molecular weight cut‐off) was inserted into the guide cannula and the probe membrane protruded 1 mm outside the guide cannula into the SCN at 8.3‐9.3 mm. Using a microinjection pump (CMA/100; CMA Microdialysis), filtered Ringer's solution (147 mm NaCl, 3.4 mm CaCl_2_, 4.0 mm KCl, pH6.0) was continuously perfused through the probe at a rate of 0.2 μL min^‐1^. The probe was connected to the microinjection pump by microbore Teflon tubing through a counterbalanced 2‐channel liquid swivel arm (Bioanalytical Systems, West Lafayette, IN, USA) attached to the rim of the bowl, thus permitting the animal to move freely without the tubing becoming tangled during the experimental period. Microdialysis samples (0.12 μL min^‐1^ flow rate) were collected into 300‐μL vials (containing 2 μL of 0.1 n perchloric acid solution) at hourly intervals through an automated refrigerated fraction collector (modified CMA/170; CMA Microdialysis) over a 24‐h period when animals were maintained on a 14 hour daily photoperiod and allowed free access to food and water.

### 2.4 High‐performance liquid chromatography (HPLC) analysis of microdialysis samples

The hypothalamic dialysis samples were analysed by HPLC with electrochemical detection (Coulochem III electrochemical detector; ESA, Chelmsford, MA, USA). The Coulochem III was equipped with a guard cell (ESA 5020; Fisher Scientific, Hampton, NH, USA) set to +350 mV and placed in‐line immediately preceding the analytical cell (Dionex, Sunnyvale, CA 5011A; E1 = −100 mV [100 μA], E2 = +225 mV [100 nA]). The stationary phase was a commercially available 150 × 2 mm column packed with C‐18 (3 μm particle size; ESA) and held at 35ºC, through which the mobile phase (MD‐TM; ESA) was forced at a rate of 0.2 mL min^‐1^. Chromatographic data were recorded by a software system (EZChrom; Agilent, Santa Clara, CA, USA) for peak integration, quantitation and analysis via external standard calibration. Standards of the neurochemicals were prepared in 0.1 n perchloric acid. The sensitivity of detection for each of the neurochemicals measured ranged from 500 fg to 1 pg. Microdialysis samples were analysed for extracellular dopamine metabolites (homovanillic acid [HVA] and 3,4‐dihydroxyphenylacetic acid [DOPAC]) (measure of dopaminergic activity)^27^, ^28^, ^29^ in the SCN and for norepinephrine (NE) metabolite (3‐methoxy‐4‐hydroxyphenylglycol [MHPG]) in the VMH.

### 2.5 Evaluation of the impact of a HFD on circadian dopamine activities in the SCN and supramammillary nucleus (SuMN) of rats

Female Sprague‐Dawley rats (14 weeks of age) were maintained on 14 hour daily photoperiods and allowed to feed and drink ad libitum. Rats were randomly divided into two groups (N = 10 per group) that were fed either a RC diet or HFD to induce weight gain (25% more than RC fed rats) for 6 weeks. A glucose tolerance test (GTT) was then performed on all rats and then, after a 3‐day rest, in vivo microdialysis was used to study daily extracellular profiles of dopamine metabolites in the SCN of rats. Microdialysis samples from the SCN of free‐living rats held under a 14 hour daily photoperiod and allowed to feed and drink ad libitum during the sampling were collected every 1 hour continuously over a 24‐hour period. Samples were assayed via HPLC for HVA and DOPAC. A sample of 5 μL from a total dialysate sample of 14 μL (ie, dialysate sample collected of 12 μL at each time point plus 2 μL of 0.1 n perchloric acid solution) was injected into the system using a refrigerated autosampler (ESA 540). At the end of the experiment, animals were perfused under anesthesia with 4% paraformaldehyde and brains were collected at 4 and 16 hour after light onset (Zeitgeber time [ZT]4 or ZT16), respectively, to quantify c‐Fos and TH double‐immunopositive neurones in the SuMN.

### 2.6 Dual‐label immunohistochemistry of c‐Fos and TH

At the end of the above‐described microdialysis experiment, HFD and RC fed SD rats were sacrificed under anesthesia by perfusion with 4% paraformaldehyde at either 5 or 16 hours after light onset (ZT5 [N_HFD _= 8; N_RC _= 8] or ZT16 [N_HFD _= 8; N_RC _= 8]), respectively, to quantify c‐Fos and TH double‐immunopositive neurones in the SuMN. The brains were similarly processed as described above (in section 2.2) for fluorogold tracing and the 30‐μm coronal sections were stored at −20°C until dual immunohistochemistry of c‐Fos and TH was performed. Briefly, the brain sections containing the SuMN were first labelled with a rabbit polyclonal anti c‐Fos antibody (dilution 1:20 000; PC38; Calbiochem Merck, Darmstadt, Germany) followed by a goat anti‐rabbit biotinylated secondary antiserum (dilution 1:500; Vector Laboratories). c‐Fos immunoreactivity was revealed as black Ni‐DAB punctates in the nucleus. Subsequently, the brain sections were labelled with the mouse monoclonal antiserum for TH (dilution 1:1000; MAB5280; Millipore) followed by a horse anti‐mouse biotinylated secondary antiserum (dilution 1:500, Vector Laboratories). TH immunoreactivity was revealed by DAB as brown staining in the cytoplasm. The c‐FOS/TH dual‐labelled cells were examined under a light microscope (Nikon). The images were captured with a digital camera and processed using photoshop without altering the ratios of signal from comparative sample regions. Dual immunopositive neurones for c‐Fos and TH at the SuMN and the adjacent posterior hypothalamus (PH) region were identified and counted manually on sequential sections across the whole SuMN in a double‐blind manner. The dual positive numbers obtained from sequential coronal sections of each brain in the same treatment group were used to generate a dual positive number per SuMN/PH test area. The between group difference in dual positive number per SuMN/PH area was then analysed by two‐way analysis of variance (anova). As a control experiment, a parallel set of coronal brain sections at the level of SuMN was processed for dopamine β‐hydroxylase (DBH) immunostaining and TH/DBH double‐immunofluorescence labelling. The coronal brain sections at the level of SuMN were labelled with rabbit polyclonal anti‐dopamine β‐hydroxylase antibody (dilution 1:2000; ab209487) followed by biotinylated goat anti‐rabbit IgG (dilution 1:500; Vector Laboratories). The signals were amplified by the ABC complex and revealed by DAB chromogen. To further confirm that TH‐immunopositive neurones detected in the SuMN are dopaminergic but not noradrenergic neurones, double‐immunofluorescence labelling were sequentially performed on the same brain section using rabbit polyclonal anti‐dopamine β‐hydroxylase antibody (dilution 1:2000; Ab209487)/DyLight 488‐conjugated donkey anti‐rabbit IgG and mouse monoclonal anti‐TH antibody (dilution 1:500; MAB5280)/RRX‐conjugated donkey anti‐mouse IgG. The TH‐immunoreactivity was detected by red RRX fluorescence and DBH‐immunoreactivity was detected by green DyLight448 fluorescence. Overlay red and green fluorescence signals showed no co‐localisation of DBH labelling with TH labelling.

### 2.7 In vivo electrophysiology recordings

Female SD rats were anaesthetised with thiobutabarbital (120 mg kg^‐1^ body weight, i.p.) and mounted in a stereotaxic apparatus. The core body temperature was maintained at 37°C with a heating pad. The skull was removed from the area overlying the right side SCN. A silver electrode was implanted at the coordinates: 1.3 mm posterior to bregma, 0.2 mm lateral and 9.2 mm ventral to the dura. The injection cannula was targeted to a region just exterior (0.25‐0.4 mm lateral) to the SCN lateral edge (peri‐SCN), whereas the electrode was placed within the SCN itself. After basal neuronal activity had stabilised, test chemicals at various doses were infused for 1 minute, with rest periods of 25 minutes between each subsequent increased dosage. Electrical signals were passed through an amplifier and surveyed using a Bio Amp ML136 (ADInstruments, Colorado Springs, CO, USA). Analyses of electrophysiological activities were conducted off line with the use of labchart, version 6 (ADInstruments) to isolate spike potentials from the background data. The dose‐response curves were analysed via the Hill equation. All data are expressed as the mean ± SEM. Statistical analysis was performed using Student's *t* test and anova to determine the treatment difference in dose‐response. A *P* < .05 value was considered statistically significant.

### 2.8 Studies on SCN neurone electrophysiological responsiveness to dopamine and dopamine receptor modulators

Female SD rats (12 weeks old) were maintained under 14 hour daily photoperiods and allowed to feed ad libitum for at least 1 week before the initiation of experimentation. To study time of day dependent differences in neuronal responses to dopamine at the SCN, electrophysiology recordings were conducted at 14 hours after light onset (ZT14) (just at onset of darkness and the onset of locomotor activity in these nocturnal rodents) or at ZT5 (sleep time of day). After basal neuronal activity had stabilised, 70 mm glutamate (loaded cannula concentration) was injected at the peri‐SCN at 14 nmol per 0.2 μL to evoke neuronal activity.^30^, ^31^ Thirty minutes after glutamate, various doses of dopamine (1, 2.5, 5, 10, 25 or 50 mm loaded cannula concentrations)^32^, ^33^ were infused (0.2‐10 nmol per 0.2 μL range in 1 minute) at the peri‐SCN each following a 1‐minute pulse of 14 nmol per 0.2 μL of glutamate infusion with rest periods of 25 minutes between each subsequent increased dosage of dopamine. Electrophysiological responses were recorded as described above. These studies revealed a marked daily variation in SCN response to peri‐SCN dopamine with an approximate 4‐fold increase in sensitivity at ZT14 vs ZT5 (see Results). Therefore, to investigate which type of dopamine receptors are involved in the SCN neuronal electrophysiological responsiveness to peri‐SCN dopamine, a series of electrophysiological experiments was conducted at ZT14 with dopamine plus either a dopamine D1 or D2 receptor antagonist or with a preferential presynaptic D2 receptor antagonist. Each experiment started with 70 mm glutamate stimulation (14 nmol per 0.2 μL), and then dopamine (5 mm) infusion (1 nmol per 0.2 μL) with and without various doses of D1 specific receptor antagonist SCH‐23390 (0, 0.06, 0.62 and 6.2 mm) (0‐1.24 nmol per 0.2 μL range) or D2 specific receptor antagonist Eticlopride (0, 0.05, 0.27, 0.53, 2.05 and 5.31 mm) (0‐1.06 nmol per 0.2 μL range) applied at the peri‐SCN in 1 minute. The SCN neuronal electrophysiological responsiveness to peri‐SCN applied dopamine (1 nmol per 0.2 μL in 1 minute) following similar glutamate stimulation was similarly studied in the presence of presynaptic dopamine D2 receptor antagonist AJ76 (9.3, 18.5 and 55.6 mm) (1.86‐11.1 nmol per 0.2 μL range in 1 minute) both with or without dopamine (5 mm) applied.

### 2.9 Direct dopamine administration to peri‐SCN

Surgery was performed on SHR rats at 16 weeks of age. Rats were anaesthetised with a mixture of ketamine/xylazine (80/12 mg kg^‐1^ body weight i.p.) and placed in a stereotaxic frame (David Kopf Instruments). A 26‐gauge guide cannula (Plastics One, Roanoke, VA, USA) was permanently implanted aimed at the lateral edge of the SCN area at the following coordinates: 1.3 mm posterior to bregma, 0.4 mm right lateral to the midsagittal suture and 7.2 mm ventral to the dura. Cannula placement was confirmed by post mortem injection of 1 μL of black ink. The guide cannula was then filled with a stylet and anchored firmly to the skull with three stainless‐steel screws and cemented in place with dental acrylic. This guide cannula allows for the subsequent peri‐SCN/SCN drug administration in unanaesthetised and freely‐moving animals. Rats were allowed to recover for 1 week following the surgery. On each treatment day, the stylets were removed and a 28‐gauge injecting cannula connected to a Hamilton syringe mounted on an infusion pump was inserted into the guide cannula. The infusion cannula was protruded 2 mm from the guide cannula. Vehicle (cerebrospinal fluid; CSF) or drug (dopamine in CSF; 5 mm) was given in a total volume of 0.2 μL with a flow rate of 0.2 μL min^‐1^ for 1 minute. A further 60 s were allowed before removing the injection cannula. The dosing (2 nmol in 0.2 μL) was chosen based upon previously published data demonstrating strong physiological responses to paraventricular nuclei (PVN) targeted administration of such dopamine dose^32^ and is in the range of endogenous dopamine content in hypothalamic regions.^33^

### 2.10 Studies on the effect of circadian‐timed dopamine administration at the SCN area on glucose tolerance and peripheral fuel metabolism

Twelve‐week‐old male SHR rats were maintained on a HFD (60% calories from fat) when held on a 14 hour daily photoperiod for 4 weeks until the SCN dopamine administration experiments began. Such rats at 16 weeks of age were continued on the HFD and a 14 hour daily photoperiod assigned to one of 2 treatment groups that were infused unilaterally at the peri‐SCN area with either vehicle (artificial CSF) or dopamine solution (2 nmol) at a rate of 0.2 μL min^‐1^ for 1 min once daily at 13 hours after light onset (ZT13) for 2 weeks. GTTs were performed after 2 weeks of treatment at 48 h prior to sacrifice for assessments of liver lipid, body fat store level, and plasma leptin and NE levels. In a subsequent study, we examined the effect of circadian timing of dopamine administration to the peri‐SCN area on glucose tolerance in SHR rats maintained on the HFD. Rats were divided into four groups and two groups of rats received dopamine (2 nmol in 0.2 μL) or vehicle at the onset of locomotor activity (ZT13), whereas two additional groups of rats received either the same dose of vehicle or dopamine infusion at the same peri‐SCN area but at ZT19 as described above. Again, GTTs were performed after 2 weeks of such treatment. At the end of the experiment, animals were killed and brains were collected and stored at −80°C for neurotransmitter analyses within the VMH and PVN.

### 2.11 VMH and PVN NE analysis from animals treated for 2 weeks with dopamine or vehicle at the peri‐SCN area at either ZT13 or ZT19

Frozen serial coronal brain sections were cut at a thickness of 300 μm on a cryostat maintained at −8°C. VMH and PVN tissues were punched out and placed in 40 μL of 2% trichloroacetic acid, sonicated and centrifuged. Supernatant was immediately analysed by HPLC with coulometric electrochemical detection (ESA) for NE and the NE metabolite MHPG content and quantified against a standard curve for each. The signal was analysed by EZChrom Elite data processing software (Agilent). Next, 10 μL of supernatant was injected into the system using a refrigerated autosampler (ESA 540). The results are expressed as pg per 10 μL of sample.

### 2.12 GTT

GTT was performed 6 h after light onset. A 50% glucose solution was administered i.p. (3 g kg^‐1^ body weight) and blood samples were taken from the tail vein before glucose administration, 30, 60, 90 and 120 min after glucose injection for plasma glucose and insulin analyses.

### 2.13 Assay of metabolic parameters

Blood glucose concentrations were determined by a blood glucose monitor (OneTouch Ultra, LifeScan, Inc.; Milpitas, CA, USA). Plasma insulin and NE were assayed by an enzymeimmunoassay using commercially available assay kits utilising anti‐rat serum and rat insulin and NE as standards (ALPCO Diagnostics, Salem, NH, USA). Liver tissue was homogenised in 5% NP‐40, heated, centrifuged and supernatant assayed for triglyceride content using a Triglyceride Determination Kit (catalogue number TR0100; Sigma‐Aldrich, St Louis, MO, USA).

### 2.14 D1 and D2 dopamine receptor autoradiography

Female SD rats weighing 225‐275 g maintained on a LD 14:10 hour daily photoperiod and fed regular rodent chow were sacrificed by decapitation, and their brains were quickly removed and frozen in liquid isopentane (−30°C) for 30 s. Coronal sections (16 μm) including the peri‐SCN and SCN regions were cut on a cryostat at −20°C and thaw‐mounted onto cold, gelatin‐coated glass slides. After drying at room temperature using an air blower, the slides were stored at −80°C in slide boxes with desiccant. Prior to using the tissue, the section mounted slides were gradually brought to room temperature. For dopamine D2 receptor binding determination, the slides were first equilibrated in Tris‐ions assay buffer (15 min at room temperature) containing 50 mm Tris‐HCl, 120 mm NaCl, 5 mm KCl, 1 mm CaCl_2_, 1 mm MgCl_2_ and 5.7 mm ascorbic acid (pH 7.4), and then incubated with dopamine D2‐receptor selective antagonist [I^125^]‐iodosulpride (approximately 0.5 nm, specific activity: 2200 Ci/mmol; NEX441; Perkin Elmer, Waltham, MA, USA) prepared in Tris‐ions assay buffer for 30 min at room temperature. The slides were drained, washed twice in ice‐cold assay buffer (2 min each) and dipped in ice‐cold water and air‐dried immediately. Nonspecific binding was evaluated by treating a parallel set of slides with the same concentration of titrated [I^125^]‐iodosulpride plus an unlabelled competitor haloperidol (10 μm) to displace [I^125^]‐iodosulpride binding. Similar procedures were implemented for D1 dopamine receptor binding of prepared brain sections. The first step was equilibration of brain sections for 15 min at room temperature in Tris‐ions assay buffer excluding ascorbic acid. The next step was incubation with dopamine D1‐receptor selective antagonist [I^125^]‐SCH23982 (approximately 0.1 nm, specific activity: 2200 Ci mmol^‐1^; PerkinElmer) prepared in Tris‐ions assay buffer in the presence of 2‐HT_2A/2C_ antagonists (50 nm ketanserin and 100 nm mianserin) for 30 min at room temperature. Finally, washes (2 min × 2) were performed in ice‐cold assay buffer, followed by dipping in ice‐cold water and air‐drying. The nonspecific binding was determined by the replacement of [I^125^]‐SCH23982 binding with a saturation concentration of unlabelled dopamine D1 selective antagonist R‐(+)‐SCH23390 (10 μm). Finally, the dried slide‐mounted tissues were exposed to a film in sealed cassette at −80°C and the autoradiography images were captured and analysed using imagej (National Institutes of Health, Rockville, MD, USA).

### 2.15 Quantitative polymerase chain reaction (qPCR) of D1 and D2 dopamine receptor mRNA expression at the SCN

Female SD rats weighing 225‐275 g , maintained on a 14:10 hour photoperiod and fed a RC diet were sacrificed by decapitation and the brains were quickly collected, frozen on dry ice and stored at −80°C. On the day of the experiment, one coronal slice (150 μm) through the medial preoptic area (mPOA) and three consecutive coronal slices (150 μm × 3) through the peri‐SCN/SCN region were cut on a cryostat at −16°C. The mPOA, combined peri‐SCN/SCN and SCN tissues were dissected with pre‐chilled brain punches (0.75 mm in diameter for mPOA and peri‐SCN/SCN; 0.5 mm in diameter for the SCN) under a dissecting microscope and immediately homogenised in ice‐cold lysis buffer. For quantification of dopamine D1 and D2 receptor levels, RNA isolation (RNAqueous, Micro Kit AM1931; Ambion Thermo Fisher, Waltham, MA, USA) and cDNA synthesis (iScript Advanced cDNA Synthesis Kit for RT‐qPCR 172‐5037; Bio‐Rad, Hercules, CA, USA) were carried out in accordance with the respective manufacturer's instructions. qPCR was performed with a Bio‐Rad SsoAdvanced Universal Probes Supermix using the Bio‐Rad Dopamine D1 receptor (DRD1) assay qRnoCEP0027016, Bio‐Rad Dopamine D2 receptor (DRD2) assay qRnoCIP0023714 and Bio‐Rad GAPDH assay qRnoCIP0050838 in accordance with the manufacturer's instructions. Matching Bio‐Rad PrimePCR templates were used to quantify the absolute amount of DRD1, DRD2 and GAPDH mRNA using icycler iq (Bio‐Rad). The dopamine D1 and D2 receptor qPCR assays were repeated using a different set of primers, reagents, PCR instrument and software. RNA was isolated with an Absolutely RNA Miniprep Kit (Agilent) and cDNA was synthesised with an AffinityScript cDNA Synthesis Kit (Agilent). Amplification was conducted with a Brilliant III SYBR green qPCR kit (Agilent) using an AriaMX instrument and analyzed with ariamx software (Agilent).

Primers:

DRD1‐F 5′‐GCGTGATCAGCGTGGACAGG

DRD1‐R 5′‐AGGGCCATGTGGGCTTTGCC

DRD2‐F 5′‐GGTGGCCACACTGGTAATGCC

DRD2‐R 5′‐CAGTAACTCGGCGCTTGGAGCT

GAPDH‐F 5′‐AACTTTGGCATTGTGGAAGG

GAPDH‐R 5′‐ACACATTGGGGGTAGGAACA

### 2.16 Statistical analysis

All data are expressed as the mean ± SEM. Statistical analysis were performed using an unpaired Student's *t* test for two group comparisons or one‐way anova for more than two group comparisons, or two‐way repeated measures anova for comparisons of treatment groups undergoing repeated measurements at different time points, as appropriate. When the overall anova result was statistically significant, a post‐hoc Dunnett's test was carried out to highlight where these differences occur. A statistical value of *P* < .05 (2‐tailed) was considered statistically significant.

## 3 Results

### 3.1 Neuroanatomical and neurophysiological studies identify dopaminergic neuronal projections from the SuMN to dopamine D2 receptor site regions within the peri‐SCN/SCN area

Our previous studies suggested a role for circadian responsiveness to systemic l‐DOPA administration in the regulation of seasonal metabolism.^7^, ^8^, ^9^, ^10^ Other studies centred on clock region neurophysiology identified a potential cause‐effect relationship between a diminution of the circadian peak in dopamine release at the general peri‐SCN region and seasonal glucose intolerance.^5^, ^6^ However, the identification of specific dopaminergic neurones projecting to a peri‐SCN/SCN neural circuit that in turn regulates peripheral metabolism has not been established. Therefore, our initial studies into a potential role of peri‐SCN/SCN circadian dopaminergic regulation of HFD‐induced insulin resistance focused on identifying the anatomy of dopaminergic neurones within the peri‐SCN region, thereby aiming to investigate a role for their circadian neurophysiology in the regulation of SCN activity and peripheral metabolism.

**Figure 1:**
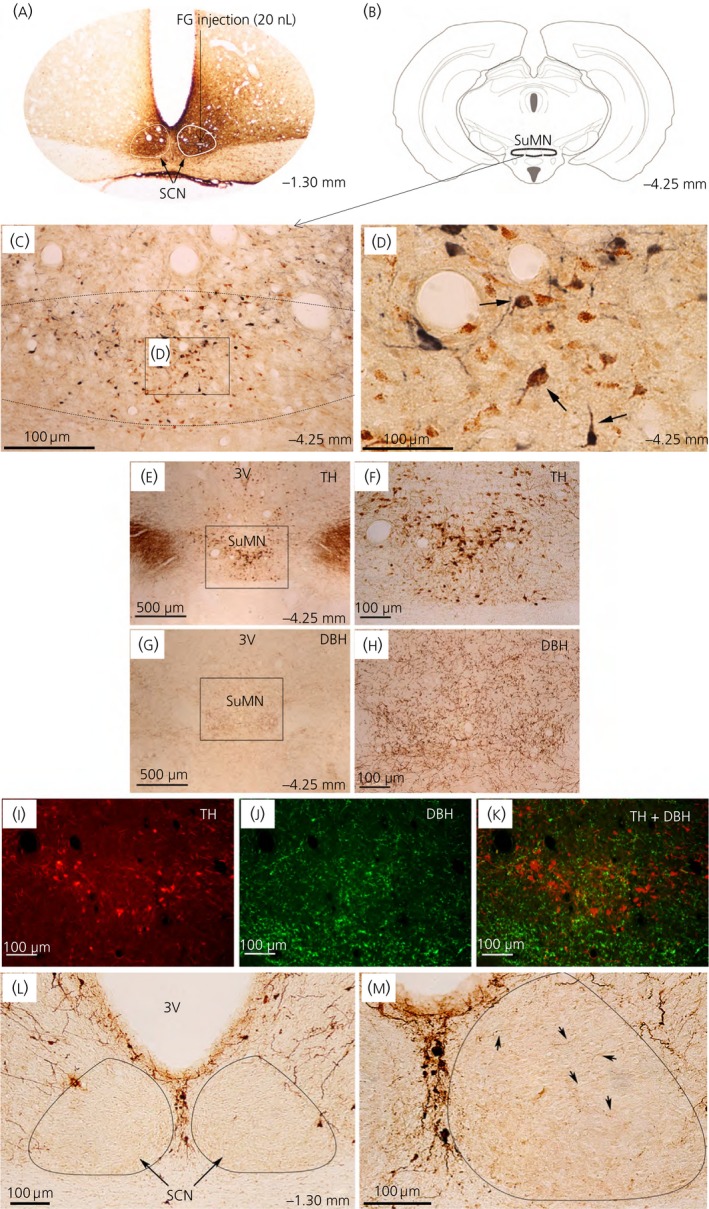
Dopamine neurones at the supramammillary nucleus (SuMN) project to the peri‐suprachiasmatic nuclei (peri‐SCN)/SCN region. (A‐D) The primary neurones that project to the peri‐SCN/SCN region were retrogradely traced by microinjection of fluorogold (FG) (4%; 20 nL) at the peri‐SCN/SCN region followed by double‐immunohistochemistry using antibodies against FG and tyrosine hydroxylase (TH) (the rate‐limiting enzyme for catecholamine synthesis). A predominant cluster of FG/TH dual immunolabelled neurones were identified at the SuMN. (A) FG at the injection site revealed by brown 3,3′‐diaminobenzidine (DAB) was localised at the peri‐SCN/SCN (Bregma ‐1.30) with diffuse transport/migration (including contralateral SCN) at 10 days after its microinjection at the right SCN. (B) Schematic of the coronal section at the level of SuMN (Bregma ‐4.25 mm). (C) FG/TH dual‐labelled cells at the SuMN (low magnification). Dashed line indicates SuMN border. (D) High magnification of FG/TH‐dual labelled cells at the SuMN. FG‐immunoreactivity was labelled as brown punctuates in the cytoplasm and processes. TH‐immunopositivity was labelled as diffuse blue‐grey staining in the cytoplasm and processes. Arrows indicate FG/TH double‐immunopositive cells. (E‐K) The TH‐immunopositive neurones that project to the peri‐SCN/SCN region are dopamine neurones. The dopaminergic neurones at the SuMN were identified as TH‐immunopostive and dopamine β‐hydroxylase (DBH)‐immunonegative neurones. TH‐immunopositive cell bodies and processes revealed by brown DAB staining using antibody against TH were found in the SuMN at low magnification (E) and at high magnification (F). DBH‐immunopositive terminals (but not cell bodies) revealed by brown DAB staining coupled with antibody against DBH were detected in the SuMN at low magnification (G) and at high magnification (H). Double‐immunofluorescence staining of TH (I) and DBH (J) on the same brain section showed no co‐localisation of TH‐immunoreactivity (red) and DBH‐immunoreactivity (green) in cells detected in the SuMN (K). Brain section: bregma ‐4.25 mm. 3V, third ventricle. (L, M) Immunohistochemical staining of dopaminergic innervations revealed by brown DAB staining coupled with antibody against TH were found predominantly in the peri‐SCN region with sparse but detectable staining in the SCN. (L) Dense TH‐immunopositive processes at peri‐SCN. (M) Sparse TH‐immunopositive processes within SCN (high magnification). Arrows point to the TH‐immunoreactivity inside SCN. Brain section: bregma ‐1.30 mm

**Figure 2:**
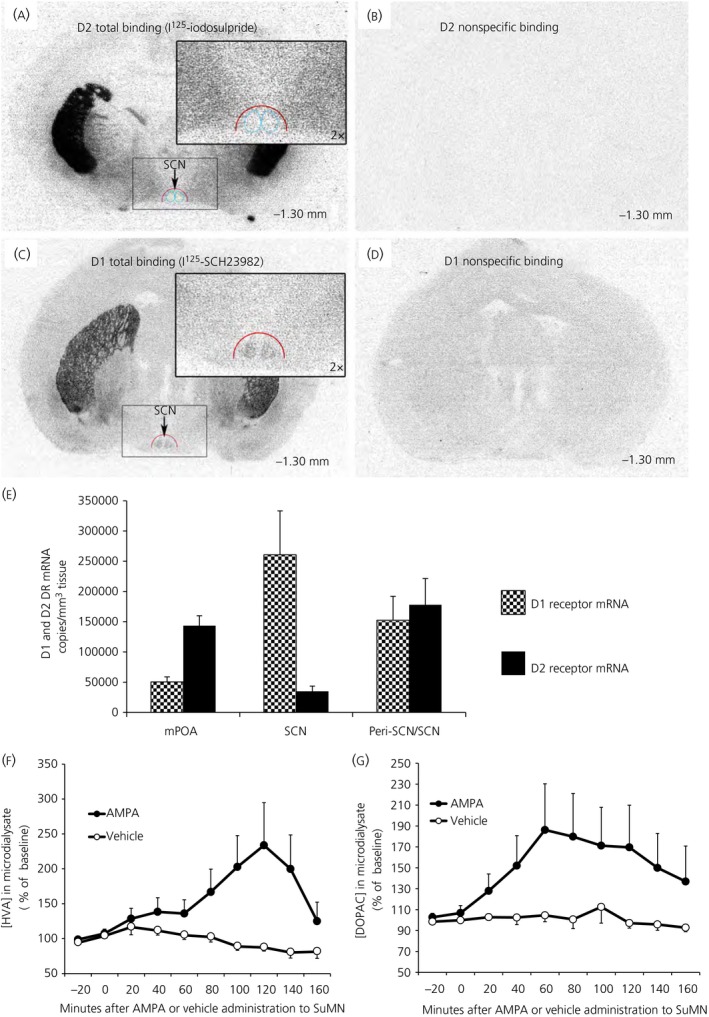
(A‐E) Dopamine receptor binding and mRNA present in peri‐suprachiasmatic nuclei (SCN)/SCN area. (A) Autoradiography using radioligands selective for dopamine receptor D2 (I^125^‐iodosulpride) in brain sections at the level of the SCN (bregma ‐1.30 mm). [I^125^]‐iodosulpride revealed low density D2 dopamine receptor binding sites within SCN (blue cycle) and higher (moderate) density binding in peri‐SCN (red semicycle). Insert: higher magnification of peri‐SCN/SCN. (B) The binding specificity of [I^125^]‐iodosulpride (0.5 nm,* K*_d_ = 1.6 nm) to D2 dopamine receptors was confirmed by the displacement of the [I^125^]‐iodosulpride binding sites with a saturation concentration of dopamine D2 receptor antagonist haloperidol (10 μm). (C) Autoradiograph ligand binding study with [I^125^]‐SCH23982 revealed a moderate density of dopamine D1 receptor binding sites within the SCN and low binding density in the peri‐SCN (red semicycle). Dopamine D1 receptors are defined by [I^125^]‐SCH23982 binding sites (0.1 nm,* K*_d_ = 0.12 nm) in the presence of 5‐HT_2A/2C_ antagonists (ketanserin, 50 nm and mianserin, 100 nm). Insert: high magnification of peri‐SCN/SCN. (D) The binding specificity to dopamine D1 receptors was confirmed by the displacement of the [I^125^]‐SCH23982 binding sites with a saturation concentration of dopamine D1 receptor antagonist R‐(+)‐SCH23390 (10 μm). (E) Dopamine D1 and D2 receptor mRNA at the medial preoptic area (mPOA), periSCN/SCN and SCN regions of the hypothalamus quantified by a quantitative reverse transcriptase‐polymerase chain reaction (PCR). Dopamine D1 and D2 receptor mRNA actual transcript number per mm^3^ of tissue at the mPOA, peri‐SCN/SCN and SCN areas were each quantified by generation of standard curves with a Bio‐Rad PrimePCR template (assay ID qRnoCEP0027016 for dopamine D1 receptor and qRnoCIP0023714 for dopamine D2 receptor) as standard. Such transcripts for dopamine D2 receptor were much lower than that at the striatum (15 million copies per mm^3^ of tissue). Relative concentrations of Dopamine D2 and D1 receptor mRNA among these brain regions were not altered when normalised to GAPDH mRNA (GAPDH quantified with Bio‐Rad assay qRnoCIP0050838). Reduction of dopamine D1 receptor mRNA transcript density in peri‐SCN/SCN vs SCN area reflects dilution of SCN transcript with peri‐SCN tissue of much reduced D1 mRNA content. Results are the mean ± SEM of tissue samples from 5 animals. (F, G) Neurophysiological dopamine communication from the supramammillary nucleus (SuMN) to SCN. Acute intra‐SuMN AMPA administration increases the extracellular dopamine metabolites 3,4‐dihydroxyphenylacetic acid (DOPAC) and homovanillic acid (HVA) at the SCN. Extracellular profiles of HVA (F) and DOPAC (G) in microdialysate samples from the SCN of freely‐moving rats that received either acute intra‐SuMN AMPA (●) or vehicle (○). Data are expressed as percentage changes from the baseline (mean ± SEM, n = 6 per group). Two‐way anova with repeated measures on HVA (F) indicates a time effect (*F*_9,90_ = 2.026, *P* < .05) and also a time and treatment interaction effect (*F*_9,90_ = 3.368, *P* <0.005). SCN DOPAC (G) is increased in AMPA treated vs vehicle groups (*F*_1,10_ = 5.387, *P* < .05). There is also a time effect (*F*_9,90_ = 2.509, *P* < .05) and a time and treatment interaction effect (*F*_9,90_ = 2.065, *P* < .05)

Neuroanatomical studies utilised nanolitre injections of the retrograde tracer fluorogold (which labels primary projecting neurone cell bodies when injected at the neuronal terminal region) at the SCN and its perimeter followed by double‐immunohistochemical staining with fluorogold and TH (the rate‐limiting enzyme in dopamine synthesis) antibodies to trace the origin of primary dopaminergic neurones that project to the peri‐SCN (the anatomical area defined as within 0.25‐0.40 mm lateral to the SCN edge)/SCN region. These studies revealed a predominant collection of TH positive (+), primary neurones within the SuMN that project to the peri‐SCN/SCN region (Figure 1A‐D). Some double‐immunopositive neurones were also detected at the posterior hypothalamic area juxtaposed to the SuMN. The SuMN area TH+ neurones were found to be DBH negative, thus identifying these neurones as dopaminergic (Figure 1E‐K). Analysis of TH+ fibre projections revealed ample projections to the peri‐SCN area, as well as few projections into the SCN itself (Figure 1L,M). Next, we identified the existence of dopamine D1 and D2 receptors in this peri‐SCN/SCN region by [I^125^]‐SCH23982 and [^125^I]‐iodosulpride autoradiography, as well as reverse transcriptase (RT)‐PCR of D1 and D2 receptor mRNA expression. Autoradiographic analysis revealed moderate dopamine D2 receptor binding density within the peri‐SCN region and much lower D2 receptor binding density within the SCN itself (Figure 2A,B), as well as moderate D1 receptor binding within the SCN and much lower D1 receptor binding density in the peri‐SCN region (Figure 2C,D). In agreement with these dopamine receptor binding data, quantitative RT‐PCR analysis with internal standards to quantify the absolute transcript number demonstrated the presence of both dopamine D2 and D1 receptor mRNA expression within the SCN, the peri‐SCN and mPOA regions (Figure 2E), with each of their quantities in each area correlating with the relative ligand binding densities of their receptors in each respective area and, overall, ranging much lower than at the striatum. The DRD1/DRD2 mRNA ratio, within and between the MPOA, SCN, and PeriSCN‐SCN regions were the same when, determined with the Bio‐Rad kits and reagents or when determined using the Agilent kits and reagents (data not shown). GAPDH measurement confirmed that RNA isolation and reverse transcription yields were comparable between samples.

In a separate study, administration of AMPA receptor agonist directly to the SuMN resulted in robust dopamine release at the SCN area (as assessed by in vivo extracellular microdialysis of HVA and DOPAC), confirming that this anatomical apparatus was functional in providing dopaminergic input to the SCN area (Figure 2F,G). Two‐way anova with repeated measures on HVA indicates a time effect (*F*_9,90_ = 2.026, *P* < .05) and also a time and treatment interaction effect (*F*_9,90_ = 3.368, *P* < .005). SCN DOPAC was increased in AMPA treated vs vehicle groups (*F*_1,10_ = 5.387, *P* < .05). There is also a time effect (*F*_9,90_ = 2.509, *P* < .05) and a time and treatment interaction effect (*F*_9,90_ = 2.065, *P* < .05).

### 3.2 The circadian peak in SuMN‐SCN dopamine release is abolished by HFD feeding

**Figure 3:**
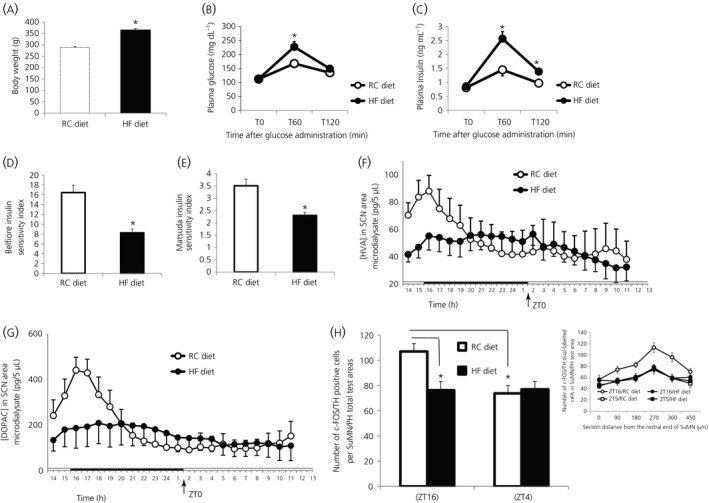
The high‐fat diet (HFD) feeding‐induced obese and insulin‐resistant condition is accompanied by a concurrent abolishment of the circadian peak in suprachiasmatic nuclei (SCN) dopaminergic activity and of the coincident daily peak in dopaminergic neurone activity in supramammillary nucleus (SuMN) neurones. Animals were fed HFD for 9 weeks and, after 32% of gain in body weight, analyses of SCN area dopamine release and SuMN dopamine activity at different circadian time points were performed. (A) Body weight of regular chow (RC, white bar) and HFD (black bar) fed rats (*P < .0001, HFD fed vs RC fed group) (Student's *t* test). Plasma glucose (B) and insulin (C) during a glucose tolerance test (*P < .05, difference between the two groups at same time) (anova with repeated measures followed by post‐hoc *t* test). The area under the glucose and insulin tolerance curve in the HFD fed group increased by 23% and 57% respectively, compared to the RC fed group (*P* < .05, Student's *t* test). HFD feeding induces insulin resistance (reduces Belfiore and Matsuda insulin sensitivity indices by 50% [D] or 34% [E], respectively, *P < .005 [Student's *t* test]). (F,G) Daily profiles of homovanillic acid (HVA) and 3,4‐dihydroxyphenylacetic acid (DOPAC), respectively in 5‐μL microdialysate samples from the SCN of freely‐moving rats fed either HFD (●) or RC (○) (n = 8 per group). The horizontal bar indicates light and dark phases of the daily photoperiod. Two‐way anova with repeated measures on HVA indicates a time of day effect (*F*_21,294_ = 4.3, *P* < .0001). There is also a time and group interaction effect (*F*_21,294_ = 3.2, *P* < .0001), which indicates a circadian difference of SCN dopamine activity between the HFD and RC fed groups. Two‐way anova with repeated measures on DOPAC (G) reveals a time of day effect (*F*_21,294_ = 5.488, *P* < .0001) and a time and group interaction effect (*F*_21,294_ = 2.578, *P* < .0002). All data are expressed as the mean ± SEM (n = 10 per group). (H) The daily peak dopamine neuronal activities at the SuMN and adjacent posterior hypothalamus (PH) were reduced by HFD feeding. The brains from HFD fed obese rats and RC fed lean rats on LD 14:10 h photoperiods were collected during the day (ZT4 [Zeitgeber time] hours after light on set) and night (ZT16), respectively. The activated dopamine neurones were identified as double immune‐positive neurones using antibodies against tyrosine hydroxylase (TH) (a rate‐limiting enzyme for dopamine synthesis) and c‐Fos (neuronal activation marker). The number of activated dopamine neurones at the SuMN/adjacent posterior hypothalamus (determined as number per total sampled areas) in the brains from RC lean rats was 46% higher at ZT16 than at ZT4 (two‐way anova analysis: *P < .05; n = 8 or 9) and this circadian peak was abolished in the brains from HFD‐fed obese rats (*P < .05). Insert: Number of double positive neurons at each sampled area within the SuMN/PH for animals within each group (mean ± SEM).

Based upon results of several previous studies demonstrating the import of (i) a circadian rhythm of responsiveness to systemically administered l‐DOPA^7^, ^8^, ^9^, ^10^ and (ii) peri‐SCN dopamine activity in the modulation of seasonal metabolism,^5^, ^6^ we postulated that these SuMN‐SCN dopaminergic neurones may represent a circadian dopaminergic circuit operative in the modulation of SCN function and its regulation of peripheral metabolism. We further postulated that this circuit may represent not only a clock mechanism regulating seasonal metabolism, but also a biological target for the fattening/insulin resistance‐inducing effects of a HFD. Therefore, our next series of studies investigated the potential existence of a daily rhythm of SuMN dopaminergic neuronal activity and a daily rhythm of dopamine release from peri‐SCN neurones among lean animals and those made obese/insulin resistant by HFD feeding. Compared to RC fed animals, HFD fed animals had increased body weight (*P* < .0001, Student's *t* test) (Figure 3A), as well as increased plasma glucose (Figure 3B) and insulin (Figure 3C) during a GTT (difference between the two groups at same time *P* < .05, anova with repeated measures followed by a *t* test). The area under the glucose and insulin GTT curve in the HFD fed group increased by 23% and 57%, respectively, compared to the RC fed group (*P* < .05, Student's *t* test). HFD feeding reduced the insulin sensitivity (ie, Belfiore and Matsuda insulin sensitivity indices by 50% [Figure 3D] and 34%, respectively [Figure 3E]) (*P* < .005, Student's *t* test). Extracellular microdialysis samples collected at 2 hour intervals over a 24 hour period from the peri‐SCN region of free living lean rats fed either RC or HFD and maintained on daily 14 hour photoperiods were subsequently analysed for dopamine metabolites. Such analysis revealed a robust circadian rhythm of dopamine release (measured as changes in extracellular levels of HVA and DOPAC) with a peak (2.5‐fold increase over the trough period, *P* < .0002) at the onset of locomotor activity (onset of darkness in these nocturnal animals; 14 hours after light onset [ZT14]) among rats fed regular chow that was completely abolished in rats fed a HFD (Figure 3F,G). Two‐way anova with repeated measures on HVA indicates a time of day effect (*F*_21,294_ = 4.3, *P* < .0001). There is also a time and group interaction effect (*F*_21,294_ = 3.2, *P* < .0001), which indicates circadian difference of SCN dopamine activity between HFD and RC fed groups. Two‐way anova with repeated measures on DOPAC reveals a time of day effect (*F*_21,294_ = 5.488, *P* < .0001) and a time and group interaction effect (*F*_21,294_ = 2.578, *P* < .0002). Furthermore, immunohistochemical staining of SuMN neurones dual positive for cFOS and tyrosine hydroxylase (ie, activated SuMN dopamine neurones) in lean animals fed low‐fat chow similarly demonstrated a time of day dependent variation in activated SuMN dopamine neurones with a peak activity at the onset of locomotor activity. The number of activated dopamine neurones at the SuMN/adjacent posterior hypothalamus in the brains from RC lean rats was 46% higher at ZT16 than at ZT4 (two‐way anova analysis: *P* < .05; n = 8 or 9 per group) and this daily peak is abolished in the brains from HFD fed obese rats (Figure 3H), thus corroborating the microdialysis study results reported above.

### 3.3 The circadian peak in electrophysiological responsiveness to dopamine at the SCN coincides with the circadian peak in dopamine release at the SCN in lean insulin‐sensitive rats and is attenuated by HFD feeding

**Figure 4:**
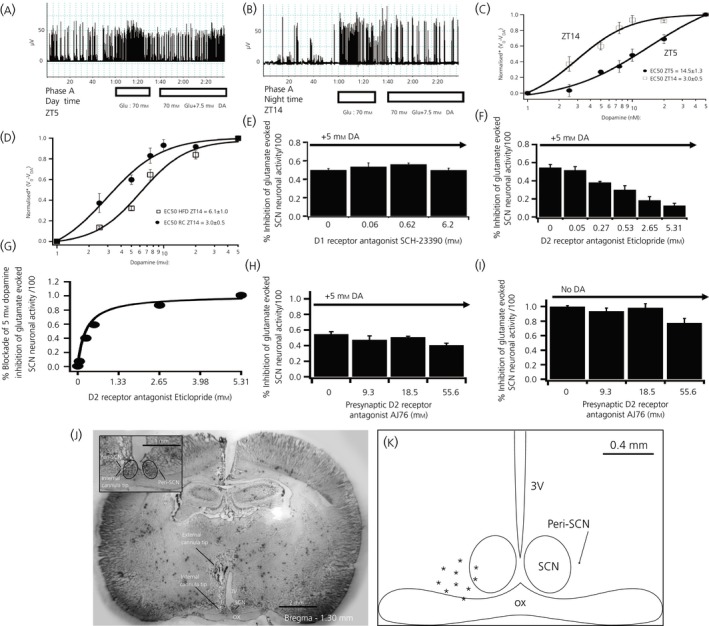
Daily peak of suprachiasmatic nuclei (SCN) neuronal electrophysiological response to dopamine coincides with the circadian peak of dopamine release at SCN in healthy insulin‐sensitive animals and is reduced in high‐fat diet (HFD) fed rats. (A, B) Electrophysiological recording example of dopamine (DA) (7.5 mM) inhibition of glutamate (70 mM) evoked SCN neuronal activity at Zeitgeber time (ZT)5 (day time) and at ZT14 (just at darkness onset and the onset of locomotor activity in these nocturnal rodents). (C) Daily variation of peri‐SCN/SCN electrophysiological sensitivity to peri‐SCN dopamine inhibition (1, 2.5, 5, 10, 25 and 50 mm) of glutamate stimulation of SCN neurones at ZT5 and ZT14. A two‐way anova with repeated measures indicates a significant difference between the SCN neuronal response to dopamine inhibition at ZT14 and at ZT5 (*F*_1,35_ = 25.597, *P* < .001). The half maximal effective concentration of dopamine inhibition to the SCN neuronal response at ZT5 (EC_50 _= 14.5 ± 1.3 mm) was 4.8 times greater than at ZT14 (EC_50 _= 3.0 ± 0.5 mm,* P* < .05). (D) SCN neuronal sensitivity response to peri‐SCN dopamine inhibition of peri‐SCN glutamate stimulation of SCN neurones at ZT14 in HFD and rodent chow (RC) fed rats. A two‐way anova with repeated measures indicates a much reduced SCN dopamine responsiveness at ZT14 in rats made obese by HFD feeding (*F*_6,48_ = 54.3, *P* < .0001). (E) SCN neuronal responsiveness to dopamine inhibition in the presence of D1 specific receptor antagonist (SCH‐23390) at ZT14. SCH‐23390 did not alter peri‐SCN glutamate evoked SCN neuronal activity responsiveness to peri‐SCN dopamine inhibition at the tested dosages. (F,G) Glutamate‐evoked SCN neuronal activity responsiveness to peri‐SCN dopamine inhibition in the presence of D2 specific receptor antagonist (Eticlopride) at ZT14. D2 antagonist dose‐dependently blocked glutamate‐evoked SCN response to peri‐SCN dopamine (*P* < .05, One way anova). D2 antagonist eticlopride EC_50_ = 265 μm. (H,I) Glutamate‐evoked SCN neuronal responsiveness in the presence of presynaptic antagonist (AJ76) at ZT14 with (H) or without (I) peri‐SCN dopamine applied. All values are the mean ± SEM (n = 5 per group). Higher dose AJ76 resulted in the slight inhibition of glutamate stimulation as may be expected from its potential consequent increase of endogenous synaptic dopamine level (subsequent to presynaptic dopamine D2 receptor blockade). (J) Histology of peri‐SCN injection cannula placement. The external cannula tip location was at the stereotaxic coordinates of anteroposterior −1.3, mediolateral 0.4 mm and dorsoventral −7.3 mm from dura. The injection cannula protruded 2 mm from the guide external cannula. The internal cannula tip shows the injection site. OX, optic chiasm; 3V, third ventricle. Enlarged peri‐SCN injection site is shown in the insert. The cannula placement was the same as the microdialysis probe location. (K) Diagrammatic representation of the peri‐SCN injection target site locations for animal brains confirmed histologically. The injection sites are between −0.1 mm and 0.4 mm lateral to the SCN. Only data from properly targeted cannula and probes were used for analyses. *Normalised *V*_0_/*V*_DA_ represents the fractional inhibition of glutamate evoked SCN neuronal activity relative to pre‐glutamate administration baseline activity level

To gain insight into a potential neurophysiological role for the circadian rhythm of dopamine release at the peri‐SCN/SCN area in the regulation of SCN neuronal activity, we next tested whether a daily variation of SCN neurone electrophysiological responsiveness to peri‐SCN/SCN area dopamine might exist in rats fed regular chow and whether this rhythmicity, if it existed, might also be disrupted by HFD feeding. In preliminary studies, peri‐SCN/SCN area dopamine administration was found to exert inhibition of neuronal firing rate of SCN neurones, whereas glutamate administration at this site potently stimulated firing rate of SCN neurones. Therefore, to assess the magnitude of peri‐SCN/SCN area dopamine inhibition on the SCN neuronal firing rate, the ability of such dopamine to inhibit glutamate‐evoked SCN neuronal activity was investigated. SCN neurones exhibited a daily variation in basal action potential activity with a peak during the daily photophase (ZT5) and little activity during the dark phase (ZT14), as established previously,^34^whereas peri‐SCN glutamate administration at 70 mm induced a marked potentiation of the SCN firing rate equally at both ZT5 and ZT14 (Figure 4A,B). Electrochemical recordings of 70 mm (14 nmol per 0.2 μL) peri‐SCN glutamate‐evoked action potentials from the SCN following administration of 5 mm dopamine directly to this peri‐SCN/SCN region demonstrated a marked inhibition in SCN firing rate in response to such dopamine administration. Moreover, in rats held on a regular chow diet, there was a robust (approximately 5‐fold) increase in sensitivity to peri‐SCN dopamine inhibition of such glutamate‐evoked SCN neuronal firing rate in response to its administration at ZT14 (a time coinciding with the daily peak in dopamine release at this area) relative to its administration at ZT5 (Figure 4C). A two‐way anova with repeated measures indicates a significant difference between the SCN neuronal response to dopamine inhibition at ZT14 and at ZT5 (*F*_1,35_ = 25.597, *P* < .001). The half maximal effective concentration of dopamine inhibition to the SCN neuronal response at ZT5 (EC_50 _= 14.5 ± 1.3 mm) was 4.8 times greater than at ZT14 (EC_50 _= 3.0 ± 0.5 mm), (*P* < .05). By contrast, among animals made obese by HFD feeding, the daily peak in SCN electrochemical responsiveness to peri‐SCN dopamine administration (at ZT14) was reduced 50% relative to RC fed controls (two‐way anova with repeated measures: *F*_6,48_ = 54.3, *P* < .0001) (Figure 4D).

Consequently, in healthy, non‐obese animals, the daily peak of dopamine release at the peri‐SCN region is “in‐phase” with the daily peak in SCN responsiveness to peri‐SCN dopamine, whereas, among animals made obese/insulin resistant by HFD feeding, these coincident daily peaks in peri‐SCN dopamine release and SCN response to peri‐SCN dopamine administration were both markedly reduced. Neuropharmacological studies were conducted to clarify the nature of the peri‐SCN dopamine receptor involved in the SCN response to peri‐SCN dopamine. Activation competition studies with peri‐SCN area administered dopamine (at the dopamine EC_50_) plus or minus dopamine D1 or D2 receptor antagonists, as well as with a selective presynaptic dopamine D2 receptor antagonist (AJ76), revealed that postsynaptic dopamine D2 receptors on peri‐SCN neuronal dendrites were responsible for mediating the SCN response (see Figure 4E‐I with legend descriptions). Verification of cannula placement used to infuse dopamine or dopamine receptor modulators to the peri‐SCN region revealed such cannula placement within 0.4 mm lateral to the SCN (Figure 4J,K).

### 3.4 Restoration of the circadian peak of dopamine at the SCN of insulin‐resistant/glucose intolerant animals held on a HFD attenuates the insulin resistance/glucose intolerance

**Figure 5:**
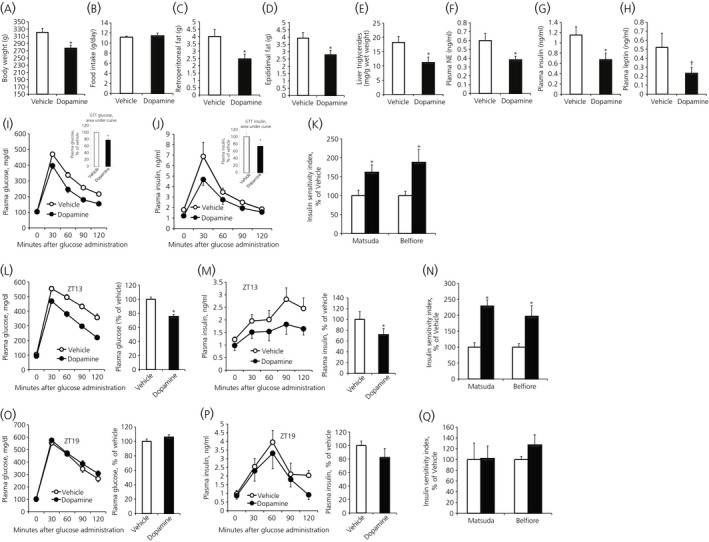
Restoring the circadian dopamine peak (Zeitgeber time [ZT]13) at the peri‐suprachiasmatic nuclei (SCN) of high‐fat diet (HFD) fed, obese insulin‐resistant spontaneously hypertensive rats (SHR) rats improves dysmetabolism without affecting food consumption, whereas the same treatment outside this circadian peak window (ZT19) has no effect on insulin resistance of SHR rats. After 6 weeks on HFD, obese animals were treated daily with either vehicle (cerebrospinal fluid) or dopamine (2 nmol) infused at the peri‐SCN for 1 min unilaterally at 13 h after light onset (ZT13) for 2 weeks when maintained on the HFD. The following metabolic parameters were assessed after 2 weeks of treatment: (A) body weight at the end of treatment; (B) daily food consumption during the treatment (no difference); (C) retroperitoneal fat pad weight; (D) epididimal fat pad weight; (E) liver triglyceride level; (F) plasma norepinephrine; (G) plasma insulin; and (H) plasma leptin (^†^ *P* < .05, 1‐tailed *t* test. (I) Glucose tolerance test (GTT) plasma glucose curves (line graph) and the area under the glucose GTT curve (bar graph). (J) GTT plasma insulin curves (line graph) and the area under the insulin GTT curve (bar graph) and (K) Matsuda and Belfiore insulin sensitivity indices of SHR rats on HFD treated with either vehicle or dopamine at ZT13. Data are expressed as the mean ± SEM (n = 9 per group) (*P < .05 compared to vehicle treatment, unpaired Student's *t* test). (L‐Q) Comparison of metabolic response to peri‐SCN dopamine administration at ZT13 vs ZT19. GTT from rats fed HFD for 6 weeks and then receiving dopamine (2 nmol) or vehicle at the onset of locomotor activity (ZT13) or at ZT19 for 2 additional weeks when held on HFD feeding. (L) GTT plasma glucose curves (line graph) and the area under the glucose GTT curve (bar graph), (M) GTT plasma insulin curves (line graph) and the area under the insulin GTT curve (bar graph) and (N) Matsuda and Belfiore insulin sensitivity indices of vehicle (white bar) or dopamine (black bar) peri‐SCN treated animals at ZT13. (O) GTT plasma glucose curves (line graph) and the area under the glucose GTT curve (bar graph), (P) GTT plasma insulin curves (line graph) and the area under the insulin GTT curve (bar graph) and (Q) Matsuda and Belfiore insulin sensitivity indices of SHR rats on HFD treated with either vehicle (white bar) or dopamine (black bar) at ZT19. Data are expressed as the mean ± SEM (n = 6 per group) (*P < .05, unpaired Student's *t* test)

We next hypothesised that the circadian peak in peri‐SCN dopaminergic activity was operative in the maintenance of normal metabolism and that the loss of such activity was permissive for the HFD‐induced obesity/insulin resistance. A series of studies were therefore conducted in animals made obese and insulin resistant by HFD feeding to examine the metabolic impact of daily dopamine administration to the peri‐SCN region made at the time of day of the natural daily peak in peri‐SCN dopaminergic activity of lean insulin‐sensitive animals. Daily administration of exogenous dopamine to the peri‐SCN region (where the SuMN dopamine terminals were observed) in animals made obese/insulin resistant by feeding of a HFD at the time of day of circadian peak dopaminergic neuronal activity at this site in healthy lean animals (onset of locomotor activity; ZT13) resulted in a substantial reduction in epididimal fat pad (29%), retroperitoneal fat pad (38%), liver triglyceride content (39%), hyperleptinaemia (55%) and hyperinsulinaemia (42%), whereas there was elevated plasma NE (36%) and improved insulin sensitivity indices (Matsuda and Belfiore indices increased by 62% and 88%, respectively) (*P* < .05, unpaired Student's *t* test for each) without any change in daily food consumption (of the HFD) (Figure 5A‐K). In a follow‐on study aiming to determine the impact of the diurnal timing of the dopamine administration to the peri‐SCN/SCN region on glucose tolerance in such HFD fed, obese/insulin‐resistant animals, peri‐SCN/SCN dopamine administration at ZT13 to such animals improved the insulin sensitivity indices (Matsuda and Belfiore indices increased by 129% and 97%, respectively) (*P* < .05, unpaired Student's *t* test for each) (Figure 5L‐N), whereas the administration of dopamine to this peri‐SCN/SCN region later during the dark phase of the day (ZT19) in such animals produced no alteration in glucose intolerance or insulin resistance and animals were noticeably ill with wasting and decreased food consumption (Figure 5O‐Q).

**Figure 6:**
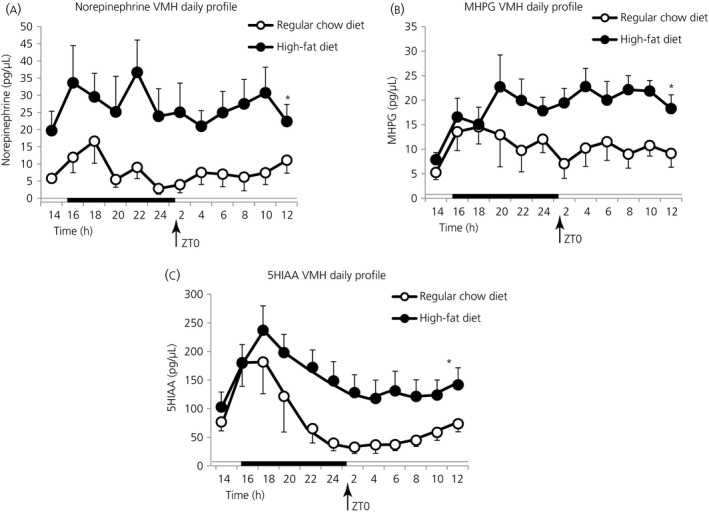
High‐fat diet (HFD) feeding induces elevations in ventromedial hypothalamus (VMH) norepinephrine and serotonin activity. Female Sprague‐Dawley (SD) rats (4 week old, n = 7‐9 per group) were fed either a HFD or rodent chow (RC) diet for 6 weeks and then 24‐h in vivo VMH extracellular profiles of monoamine metabolites were investigated by 24‐h microdialysis sampling in each group. Daily profiles of (A) NE, (B) MHPG and (C) 5‐HIAA in microdialysate samples from VMH of freely‐moving rats fed either HFD (●) or RC (○) (n = 7 or 8 per group). The horizontal bar indicates light and dark phases. HFD fed rats exhibited elevated levels of VMH 3‐methoxy‐4‐hydroxyphenylglycol (MHPG), norepinephrine (NE) and 5‐HIAA compared to RC diet rats (by 38%, 63% and 48%, respectively). Repeated measures anova on NE indicated a group effect (*F*_1,13_ = 14.01, *P* < .005), as did repeated measures anova on MHPG (*F*_1,13_ = 5.334, *P* < .05). Repeated measures anova on 5‐HIAA also revealed a group effect (*F*_1,13_ = 7.630, *P* < .05)

**Figure 7:**
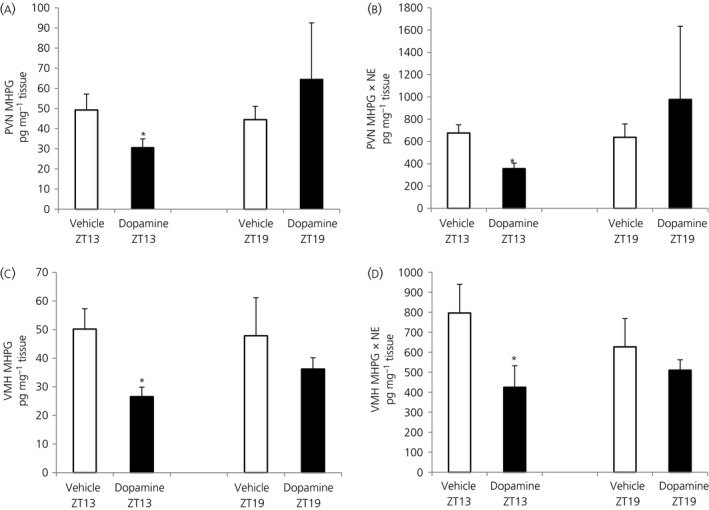
Dopamine administration at the peri‐suprachiasmatic nuclei (peri‐SCN) at Zeitgeber time (ZT)13 in high‐fat diet (HFD) fed rats to restore the normal circadian peak of dopamine at this site but not when administered outside this circadian peak window (at ZT19) reduced noradrenergic turnover/activity (3‐methoxy‐4‐hydroxyphenylglycol [MHPG] and MHPG × norepinephrine [NE] product) in both the ventromedial hypothalamus (VMH) and paraventricular nuclei (PVN). (A) PVN MHPG, (B) PVN MHPG × NE, (C) VMH MHPG and (D) VMH MHPG × NE content (pg mg^‐1^ tissue) in rats receiving dopamine (2 nmol) or vehicle infusion into the peri‐SCN area at the onset of locomotor activity (ZT13, n = 9 per group) and in rats receiving the same dose of vehicle or dopamine infusion at ZT19 (n = 7 or 5, respectively). Data are expressed as the mean ± SEM. (*P < .05 compared to vehicle treatment, unpaired Student's *t* test)

To explore possible neuronal circuits involved in the beneficial metabolic response to peri‐SCN/SCN dopamine release in obese/insulin‐resistant animals, we next investigated the potential impact of such peri‐SCN/SCN dopamine administration on VMH and hypothalamic PVN noradrenergic activity. The rationale for such an exploration was based upon several studies establishing a cause‐effect relationship between elevated noradrenergic activity at these nuclei and dysmetabolism.^2^, ^5^, ^35^, ^36^, ^37^ Importantly, in the present study, among rats made obese and insulin resistant by HFD feeding, an increased daily level of noradrenergic (extracellular MHPG and NE) and serotonergic (extracellular 5‐HIAA) activity at the VMH was observed (38%, 63% and 48%, respectively) (*P* < .05, *P* < .005 and *P* < .05, respectively) (Figure 6). Repeated measures anova on NE indicated a group effect (*F*_1,13_ = 14.01, *P* < .005), as did repeated measures anova on MHPG (*F*_1,13_ = 5.334, *P* < .05). Repeated measures anova on 5‐HIAA also revealed a group effect (*F*_1,13_ = 7.630, *P* < .05). Following 2 weeks of infusion of dopamine or vehicle into the peri‐SCN/SCN region of obese/insulin‐resistant rats at either ZT13 or ZT19 and subsequent to death, brain punches of frozen sections of the hypothalamus were obtained for the analysis of VMH and PVN NE and its metabolites within these study groups. It was found that daily dopamine infusion at the peri‐SCN area at ZT13 (the daily peak in dopaminergic activity at this site) but not at ZT19 significantly reduced NE activity (measured as total NE content and NE × NE metabolite product in both the VMH [by 46% and 47%, respectively; *P* < .05] and the PVN [by 33% and 43%, respectively; *P* < .05]; unpaired Student's *t* test) (Figure 7).

## 4 DISCUSSION

These studies demonstrate the functionality of a unique, potent and previously unrecognised role for the circadian organisation of dopaminergic activity at the peri‐SCN/SCN neuronal area in the regulation of peripheral metabolism and of the responsiveness to the deleterious metabolic effects of a HFD. This circadian physiological activity is characterised by a temporal interaction of a circadian rhythm of dopamine release at this peri‐SCN/SCN site from SuMN neurones with a circadian rhythm of SCN neuronal electrophysiological responsiveness to such dopamine release. The SuMN is a heterogeneous neuronal locus involved in regulation of reward/primary reinforcement, hippocampal theta rhythm and arousal response; however, the present study is the first report of SuMN dopaminergic neurones projecting directly to the peri‐SCN/SCN area. The SuMN dopamine neurones also project to the lateral septum and MPOA,^38^, ^39^ both of which have strong neuronal communications with the SCN.^40^, ^41^, ^42^ In lean insulin‐sensitive animals held on a low‐fat diet, the circadian rhythm of dopamine release at the peri‐SCN/SCN region is in‐phase with the circadian rhythm of SCN neuronal responsiveness to such peri‐SCN dopamine, with both events peaking at the onset of locomotor activity (ZT13‐14). Contrariwise, in animals made obese/insulin resistant by HFD feeding, the peaks in circadian rhythms of both dopamine release and dopamine response at the peri‐SCN and SCN region, respectively, are markedly attenuated. Reinstatement of the circadian peak (ZT13‐14) in extracellular dopamine at this peri‐SCN/SCN site in such HFD fed, obese/insulin‐resistant animals by appropriately time‐pulsed administration (at ZT13) of exogenous dopamine reversed the obese, insulin‐resistant, glucose intolerant condition without affecting food consumption when animals remained on the HFD. Although this acute 1‐minute pulse of dopamine may not precisely mimic the temporal architecture of the endogenous circadian acrophase of dopamine release at this site in healthy animals (Figure 3F,G), it was sufficiently similar to such to re‐establish the normal metabolism associated with its normal daily peak release in healthy animals. Importantly, this effect of exogenous dopamine administration to the peri‐SCN/SCN area on impaired glucose tolerance was absent when administered outside of the circadian peak “window” in peri‐SCN/SCN dopaminergic activity (ZT19) observed in healthy, glucose tolerance animals at ZT13. Collectively, these findings suggest that the circadian peaks of peri‐SCN/SCN dopamine stimulus and response rhythms modulate the SCN regulation of peripheral fuel metabolism.

Receptor activation competition studies indicate that the peri‐SCN/SCN dopamine electrophysiological response system is mediated by a post‐synaptic dopamine D2 receptor in the peri‐SCN/ SCN region. Moreover, in this peri‐SCN/SCN region, qPCR identified dopamine D2 receptor mRNA and in situ autoradiographic binding studies identified functional dopamine D2 receptors both with predominance in the peri‐SCN region. Furthermore, ample TH+ fibres from the SuMN (ie, that were identified as dopaminergic) were identifiable in this peri‐SCN region and their sparse presence was even identifiable in the SCN itself. Some peri‐SCN dopaminergic neurones were found to originate from the posterior hypothalamus as well, and it cannot be ruled out that some peri‐SCN TH+ neurones represent short interneurones at the SCN perimeter with the employed technique. Such observations are in agreement with and also add site of origin information to the previous identification of TH+ dopaminergic neurones in this peri‐SCN/SCN region of adult rodents.^11^, ^12^, ^13^, ^14^, ^15^, ^16^, ^17^, ^18^ Although dopamine D1 receptor mRNA and binding were negligible within the peri‐SCN region and D1 receptor antagonists did not modify the SCN electrophysiological response to peri‐SCN dopamine, we did observe moderate D1 receptor mRNA level and binding within the SCN itself, in agreement with other such reports.^43^, ^44^, ^45^, ^46^ This is intriguing because (i) a recent study in adult rats surprisingly identified a role for SCN dopamine D1 receptor activation in the rate of entrainment to shifts in photoperiod,^47^ counter to previously held assumptions that such SCN dopamine D1 receptor activated effects were restricted to the foetal and neonatal periods;^48^, ^49^, ^50^, ^51^ (ii) in other neural centres, activation of dopamine D1 receptor containing neurones local to dopamine D2 receptor containing neurones has been shown to augment the D2 receptor activation response;^52^, ^53^ and (iii) we have observed a strong positive (even synergistic) interaction between dopamine D2 and D1 receptor agonist systemic co‐administration on amelioration of insulin resistance syndrome in rodents.^54^, ^55^, ^56^, ^57^ Although speculative at this time, it may be that circadian time‐dependent dopamine D1 receptor stimulation within the SCN contributes to the circadian metabolic responsiveness to dopamine administration at the peri‐SCN/SCN region, either associated or not with dopamine D2 receptor activity (ie, paracrine effects).^58^, ^59^, ^60^ The present findings suggest that dopamine D2 receptor activation of peri‐SCN/SCN neurones influences (directly and/or indirectly) SCN functions that regulate peripheral fuel metabolism. Finally, it must be appreciated that some aspect of the peri‐SCN dopamine stimulus‐response system may exert influence on peripheral fuel metabolism independent of action on SCN neurones. Further studies of local SCN dopamine, dopamine D2 receptor agonist and dopamine D1 receptor agonist administration to insulin‐resistant, SCN‐lesioned animals are required to test the above mentioned possibilities and we are undertaking such investigations. Moreover, our future studies aim to delineate the specifics of the phenotype of these dopamine‐sensitive peri‐SCN neurones.

Subsequent to our initial postulate that temporal interactions of circadian neuroendocrine input activity rhythms to the biological clock modulate its output signals that regulate peripheral metabolism,^1^, ^2^, ^9^, ^10^, ^61^, ^62^ a multitude of studies have provided evidence supporting such a regulatory role for the SCN in the modulation of peripheral fuel metabolism.^63^, ^64^, ^65^, ^66^ First, complete destruction of the SCN was shown to lead to insulin resistance, glucose intolerance and weight gain, clearly identifying a functional SCN as necessary to maintain normal fuel metabolism.^67^ However, the results from more detailed investigations of the role of the SCN in the regulation of peripheral fuel metabolism ascribe major roles for specific temporal interactions of specific circadian input signals to the SCN clock system with respect to directing its regulation of metabolism. The SCN sends direct and indirect projections to many hypothalamic centres that modulate peripheral metabolism, including the VMH, PVN, dorsal medial hypothalamus, lateral hypothalamus and arcuate nuclei, and also to behavioural/feeding centres in the mesolimbic system.^41^, ^42^, ^68^, ^69^ With regard to the present study, the construct of this postulate is that the circadian dopaminergic message at the peri‐SCN/SCN area is integrated within the SCN clock system with other local environmental information to modulate SCN output signalling to other downstream metabolic regulatory sites including but not limited to noradrenergic activity at the VMH and PVN activity, each of which in turn regulates peripheral metabolism, in part by adjusting phase relations of numerous metabolic circadian stimulus and response rhythms in target tissues (eg, circadian rhythm of plasma insulin stimulus interacting with a circadian rhythm of hepatic lipogenic responsiveness to insulin).^1^, ^2^, ^3^, ^61^

The present studies suggest that a contributing mediator of this circadian peri‐SCN dopaminergic effect on metabolism likely includes SCN communication with the VMH and PVN, where elevated levels of NE (observed to be present in the VMH of HFD fed animals in the present study) (Figure 6) are reduced towards normal following peri‐SCN dopamine administration. The neuronal connections from the SCN to the VMH and PVN include its indirect projections to the brain stem^70^, ^71^, ^72^, ^73^, ^74^ where noradrenergic nuclei provide the majority of noradrenergic input to the VMH and PVN.^75^, ^76^, ^77^, ^78^ The SCN also sends strong projections directly to the PVN^41^, ^79^, ^80^, ^81^, ^82^ a neural centre with strong regulatory control over peripheral metabolism.^83^, ^84^

In seasonally obese/insulin‐resistant animals, the diminution of the circadian peak of dopaminergic activity at the peri‐SCN is coupled with substantial increases in NE release at the VMH.^5^, ^35^ Exogenous infusion of noradrenaline into the VMH of seasonally lean/insulin‐sensitive animals induces the obese/insulin‐resistant condition within just a few weeks without any alteration in the food consumption of a low‐fat diet.^36^ Similarly, elevations of VMH noradrenergic activity have been consistently documented in a wide variety of other animal models of insulin resistance, including *ob/ob* mice, *db/db* mice, *A* ^*y*^ */J* mice, offspring of malnourished mother rats and offspring of insulin‐treated mother rats.^2^

Moreover, infusion of NE into the VMH of lean/insulin‐sensitive inbred laboratory rats held on a low‐fat diet to raise the extracellular NE levels induces a rapid and sustained (chronic) simultaneous increase in sympathetic nervous system (SNS) tone, plasma NE, blood pressure, insulin, glucagon and leptin levels, as well as adipocyte lipogenic activity and adipocyte responsiveness to the lipolytic effects of noradrenergic stimulation (resulting in increased free fatty acid [FFA] release that potentiates insulin resistance in liver and muscle^37^). This VMH NE effect ultimately leads to the obese, insulin‐resistant, glucose intolerant, leptin resistant and hypertensive state without altering food consumption.^36^, ^37^, ^85^ Increased VMH NE activity also results in a loss of appropriate fuel (FFA and glucose) sensing by these VMH neurones, such that, instead of responding to increased meal‐time FFA and glucose by sending neuroendocrine signals to increase peripheral insulin sensitivity as would normally occur, the NE‐overstimulated VMH now sends neuroendocrine signals to the peripheral tissues that counter insulin action in liver and muscle.^2^, ^86^ At the PVN, increased noradrenergic activity potentiates the release of both neuropeptide (NPY) and corticotrophin‐releasing hormone (CRH),^87^, ^88^, ^89^, ^90^, ^91^ that in turn can facilitate increased SNS tone, hypothalamic‐pituitary axis activity, and thereby insulin resistance, at the same time as inducing hyperinsulinaemia at the β‐cell (to facilitate fattening), synergising with increased VMH NE activity to precipitate the insulin resistance syndrome.^54^ Additionally, direct SCN GABAergic and glutaminergic projections to the PVN have been shown to influence this SNS output to the liver and regulate the daily variation in glucose tolerance and liver glucose output in rodents.^82^, ^92^, ^93^ The results of the present study indicate that peri‐SCN/SCN processing of circadian dopaminergic input signals regulates the magnitude and nature of prandial metabolism (levels of hyperinsulinaemia, hepatic lipid storage, glucose disposal, etc.) in part via peri‐SCN/SCN output modulation of such VMH and PVN functions. Therefore, dopaminergic activation of the peri‐SCN/SCN consequently drives reductions in overactive noradrenergic input activity to the VMH and PVN, which in turn can reduce neuroendocrine signals that potentiate the insulin resistance syndrome, including (i) overactive sympathetic outflow to the liver, adipose and vasculature to counter hepatic and muscle insulin action; (ii) overstimulation of glucagon secretion; (iii) overactive PVN CRH release and overactive hypothalamic‐pituitary‐adrenal axis; (iv) overactive PVN NPY release potentiating increased SNS tone and hyperinsulinaemia; (v) leptin resistance leading to dysglycaemia; and (vi) loss of appropriate hypothalamic fuel sensing,^2^, ^86^ thereby ameliorating the insulin‐resistant syndrome. Clearly, other SCN‐output neuroendocrine mediators are likely involved in this daily peak dopamine‐SCN clock circuit regulation of metabolism in that the SCN communicates directly and indirectly with several other central nervous system sites besides the VMH and PVN, which have regulatory control of peripheral fuel metabolism.^41^, ^81^, ^94^, ^95^

This peri‐SCN/SCN‐targeted dopaminergic treatment reduced both body and liver fat without any alteration of the consumption of a HFD, suggesting an induced increase in metabolic rate and/or reduction of de novo lipogenesis, and both such alterations have been observed in response to circadian timed systemic dopamine agonist treatment of insulin‐resistant animals.^4^, ^55^, ^96^, ^97^ Moreover, both systemic and peri‐SCN directed dopamine agonist treatment reduced hyperinsulinaemia (an effect that would lead to a reduced lipogenic rate). The reduction in lipogenesis observed with systemic dopamine agonist administration was coupled with a substantial increase in the protein turnover rate,^96^ and such an action is a major contributor to the resting metabolic rate. If adipose and non‐adipose tissue lipid stores are reduced as a result of energy flux away from lipid accretion and towards protein turnover, the adverse impact of the HFD on both body fat store level and insulin sensitivity (eg, via the facilitation of lipotoxicity^98^, ^99^, ^100^) may be expected to be lessened, as observed in the present study. Such systemic dopamine agonist treatment also reduced elevated sympathetic tone, hypertension, hyperleptinaemia, and hepatic inflammatory and gluconeogenic activity,^97^ consequently reducing multiple components of insulin resistance syndrome.

It is clear that it is the circadian rhythm of peri‐SCN dopaminergic activity and not merely the absolute level of such activity that is critical in manifesting the attenuation of the insulin‐resistant/glucose intolerant state because the addition of dopamine to this site at the time of its normal circadian peak in insulin‐sensitive/glucose tolerant animals (but not at a time outside this daily) interval manifests this physiological response. It is equally instructive that the impact of the HFD feeding to reduce peri‐SCN/SCN dopamine activity was restricted to the circadian peak dopaminergic activity period of the day at this site and showed no such effect during the long low trough activity period of the day (Figure 3), implicating an impact on a circadian coupling/expression mechanism and not merely comprising a biochemical inhibitory phenomenon.

Important unanswered questions of the present study include: (i) what is the genesis of these circadian dopamine stimulus and response rhythms at the clock (ie, does the circadian rhythm of SuMN dopamine release at the SCN derive from entrainment by the SCN itself and/or from other sources such as the gut‐brain axis?) and (ii) how does HFD feeding reduce the concurrent circadian peaks in SuMN‐SCN tyrosine hydroxylase activity and dopamine release, as well as SCN responsiveness to peri‐SCN dopamine stimulation? However, the finding of reduced dopaminergic activity is generally consistent with a multitude of studies indicating a reduction of dopamine and/or dopamine receptor levels in other brain areas, particularly the striatal‐mesolimbic system, following chronic HFD feeding, although no specific investigation of such feeding on circadian aspects of dopaminergic activity at these brain sites has been investigated.^19^, ^20^, ^21^, ^22^, ^23^, ^24^, ^25^, ^26^, ^101^, ^102^ Interestingly, striatal reduction of dopamine levels may partly be the result of reduced gut synthesis of the diet‐derived satiety factor, oleoylethanolamine, following HFD feeding, the gastric presentation of which is known to inhibit dopamine efflux in the striatum via vagal inputs.^103^ Although attenuation of dopamine function within the mesolimbic system has been associated with a reduction in appropriate reward signalling and consequent overfeeding as a compensatory response to chronic reduced dopaminergic signalling, the presently described circadian dopamine‐SCN clock system for regulation of glucose metabolism and body fat appears to be quite a different aspect of CNS dopamine regulation of metabolism in that its influences to attenuate the obese/insulin‐resistant state do not require and are independent of a reduction of feeding in animals held on a HFD. The present findings suggest that this circadian dopamine‐SCN clock regulatory pathway may be an important modulator of sensitivity to the metabolic effects of a HFD (ie, inducing a HFD sensitive vs HFD resistant phenotype) dependent upon the circadian nature of the dopamine input message to the SCN clock system.

Although this circadian dopamine input signalling system to peri‐SCN/SCN neurones to regulate clock functions controlling peripheral fuel metabolism is a unique finding, it mirrors the similar circadian dopamine regulation of striatal clock gene expression.^104^, ^105^ In the striatum and several other areas of the brain, circadian dopamine‐dopamine receptor interactions regulate the circadian expression of cellular clock genes that in turn modulate (i) particular functionalities of the neurone, as well as (ii) the dopamine‐dopamine receptor circadian interaction (ie, feedback).^106^, ^107^, ^108^, ^109^ The circadian dopamine‐dopamine receptor interactions at the peri‐SCN/SCN may well function to regulate circadian dopamine‐dopamine receptor interactions governing clock gene expression in the striatum in that the SCN has been observed to modulate striatal circadian neuronal activities.^68^, ^110^ This circuit may have major implications for linking visceral metabolism and behaviour as discussed below.

Neuroanatomical studies indicate that the major source of the peri‐SCN dopamine derives from the SuMN. The SuMN is a hypothalamic nucleus with connections to widespread regions of the hippocampus, forebrain areas, raphe nuclei and limbic system areas.^38^ Its role has thus far been defined as an integration centre for cognitive and emotional aspects of behaviour, including reward functions of the nucleus accumbens.^38^, ^111^, ^112^ The SuMN contributes greatly to theta rhythm generation in the hippocampus^38^ and such a general pacemaker role for synchronising neuronal activity may apply to other efferent projections including to the SCN. However, a role for the SuMN in regulating peripheral fuel metabolism or as a regulator of SCN function has not been delineated previously. Because the SuMN modulates cognitive, emotional and reward functions, a connection to metabolic regulatory centres in the hypothalamus makes the SuMN a unique relay centre for the integration and regulation of cognitive/emotional/reward functions with metabolic activity (ie, potential role in stress induced feeding changes and metabolic shifts). AMPA stimulation of the SuMN results in dopamine release at the nucleus accumbens where dopamine functions as the molecular signal of reward and we have identified an AMPA stimulatory effect on SuMN dopamine release at the SCN in the present study. If a loss of SuMN driven dopamine release at the nucleus accumbens leading to overfeeding to achieve reward is concurrent with a diminution of its circadian peak dopamine release at the SCN that facilitates insulin resistance, then these events would synergise to potentiate obesity. Indeed, in preliminary studies, we have observed that attenuation of overall SuMN activity (with GABA agonist, glutamate antagonist cocktail) resulted in overfeeding, obesity and insulin resistance/glucose intolerance within a couple of weeks.^113^

In conclusion, the present study has identified a previously unrecognised circadian dopaminergic neuronal input modulator system to the biological clock comprised of a circadian rhythm of dopamine release at the peri‐SCN area from SuMN neurones and a circadian rhythm of SCN neurone electrophysiological responsiveness to such dopamine release (at the dopamine D2 receptor) for which temporal interaction is coupled with the regulation of peripheral fuel metabolism without inducing a change in daily food consumption. In lean animals, the circadian peaks of these dopaminergic stimulus and response rhythms (are programmed to) coincide, whereas, in animals made obese/insulin resistant by HFD feeding, the daily peaks in these circadian rhythms of both dopamine release and electrophysiological response to dopamine at the SCN are markedly attenuated. The reversal of this attenuated circadian peak in dopamine signal at the peri‐SCN/SCN of HFD‐induced obese/insulin‐resistant animals by the appropriately circadian timed daily 1‐minute pulse infusion of dopamine directly to the peri‐SCN area reverses the obese, insulin‐resistant, glucose intolerant state, without altering food consumption, whereas dopamine treatment outside this circadian temporal window is ineffectual with respect to producing this metabolic response. This circadian‐time dependent effect of dopamine input activity at the SCN to improve the obese/insulin‐resistant condition involves a reduction in noradrenergic activity at the VMH and PVN of the hypothalamus, comprising areas where increased noradrenergic activity has been shown to induce the obese insulin‐resistant state even in animals fed a low‐fat diet. Normal activation of this circadian dopaminergic clock regulatory circuit appears to set the responsiveness to the adverse effects of a HFD because its activation maintains the lean/insulin‐sensitive state even in the presence of a prolonged exposure to the HFD without any alteration of feeding. Modulation of a disrupted circadian dopaminergic neuroendocrine regulation of metabolism may be an effective means of treating a host of metabolic disorders.

## CONFLICTS OF INTEREST

All authors are current or former employees of VeroScience LLC.
